# First report on molecular identification of *Anisakis simplex* in *Oncorhynchus nerka* from the fish market, with taxonomical issues within Anisakidae

**DOI:** 10.21307/jofnem-2021-023

**Published:** 2021-03-26

**Authors:** Alina E. Safonova, Anastasia N. Voronova, Konstantin S. Vainutis

**Affiliations:** 1 Far Eastern Federal University, Sukhanova Street, 8, Vladivostok, Russia; 2 Federal Scientific Center of the East Asia Terrestrial Biodiversity FEB RAS, pr. 100-letija, 159, Vladivostok, Russia

**Keywords:** Detection, Anisakidosis, *Anisakis simplex*, *Oncorhynchus nerka*, Systematics, Taxonomy, ITS, *Skrjabinisakis*, *Peritrachelius*

## Abstract

Alive anisakids cause acute gastrointestinal diseases, and dead worms contained in food can provoke sensibilization and allergic reactions in humans. Detected in the purchased minced salmon *Oncorhynchus nerka* nematodes were identified as *Anisakis simplex sensu stricto* (Anisakidae). We found that recently published phylogenetic trees (reconstructed using different ribosomal and mitochondrial genetic markers) showed independent clusterization of species recognized in the *A. simplex sensu lato* species complex. This prompted us to undertake this full-fledged molecular genetics study of anisakids from Kamchatka with phylogenetic reconstructions (NJ/ML) and calculated ranges of interspecific and intergeneric p-distances using ITS1-5.8S-ITS2 sequences. We confirmed that molecular markers based on the ITS region of rDNA were able to recognize ‘pure’ specimens belonging to the cryptic species. We offer new insights into the systematics of anisakids. The genus *Anisakis sensu stricto* should include *Anisakis simplex sensu stricto*, *Anisakis pegreffii*, *Anisakis berlandi*, *Anisakis ziphidarum*, and *Anisakis nascettii*. Presumably, two genera should be restored in the structure of the subfamily Anisakinae: *Skrjabinisakis* for the species *Anisakis paggiae*, *Anisakis brevispiculata*, and *Anisakis physeteris*; and *Peritrachelius* for the species *Anisakis typica*. In addition, we provide the short annotated list of some genera of the family Anisakidae, including their diagnoses.

Commercial fish species *Oncorhynchus keta* (Walbaum, 1792), *O. nerka* (Walbaum, 1792), *O. tshawytscha* (Walbaum, 1792), *O. kisutch* (Walbaum, 1792), *Gadus morhua* (Linnaeus, 1758), *Clupea harengus* (Linnaeus, 1758), *Merluccius merluccius* (Linnaeus, 1758), anchovy (*Engraulis* sp.), sardine (*Sardina* sp.), *Scomber* sp. from the Atlantic and Pacific Oceans are potentially susceptible to infection by third-stage larvae (L3) of anisakids. Thus, up to 98% of the scomber and 94% of the cod (*G. morhua*) in the Asian food markets were infected (Setyobudi et al., 2011); almost 34% of fish (mackerel, hake, scomber) from the Atlantic and Mediterranean Sea are reservoir hosts for *Anisakis* spp. (Debenedetti et al., 2019). In the north of Primorsky Region (Russia) infection intensity by *Anisakis* spp. of chum salmon (*O. keta*) and herring (*C. pallasii* Valenciennes, 1847) is very high (95%) (Kravtsova et al., 2004; Rybnikova et al., 2009). Along with fish, the larvae of anisakids commonly parasitize the edible parts of squids. Larval stages of three species of the genus *Anisakis* (Dujardin, 1845): *A. simplex* (Rudolphi, 1809), *A. pegreffii* (Campana-Rouget and Biocca, 1955), and A*. physeteris* (Baylis, 1923) are the causative agents of human gastrointestinal anisakidosis (Karmanova, 2007). The whole anisakids and decomposed worms in thermally processed food can provoke severe allergic reactions range from hives and angioneurotic edema to anaphylactic shock (Audicana et al., 2002; Hoshino and Narita, 2011). Anisakidosis is a common disease in Holland, Germany, France, Italy, Spain, Greece, USA, countries of the Pacific coast of Latin America, China, Japan, Thailand, in the Russian Far East; Japan has the highest incidence (up to 1,000 cases per year) (Baird et al., 2014; Nieuwenhuizen and Lopata, 2014; Pravettoni et al., 2012; Yera et al., 2018). In Russia, the problem of anisakidosis remains insufficiently studied. There are several studies of the extensiveness and intensity of fish invasion (Pacific herring, chum salmon, pink salmon, cod, flounder, greenling, mackerel, hake, saury, sea bass, smelts) and the assessment of the viability of nematode larvae (Besprozvannykh et al., 2003; Kravtsova et al., 2004; Rybnikova et al., 2009; Vyalova, 2002). Four cases of *A. simplex* larvae were described in the human stomach (Kravtsova et al., 2004; Solovieva and Taran, 2000).

In current taxonomy there is a complex of species *A. simplex sensu lato* (Mattiucci et al., 1997; Nascetti et al., 1983, 1986) which is made up of the three sibling species: *A. simplex sensu stricto*, *A. pegreffii*, and *A. berlandi* (Mattiucci et al., 2014). But, according to the available literature data a clear division of species (*A. simplex*, *A. pegreffii*, and *A. berlandi*) has been repeatedly demonstrated on phylogenetic trees reconstructed using nuclear ITS rDNA (Pekmezci et al., 2014; Tunya et al., 2020) and mitochondrial *cox2* gene (Anshary et al., 2014; Barcala et al., 2018; Mattiucci et al., 2009; Setyobudi et al., 2011; Valentini et al., 2006) DNA markers, and finally complete mitochondrial genomes (Yamada et al., 2017). Also *A. typica* (Diesing, 1860) is suspicious with its uncertain position on different phylogenetic reconstructions (Iñiguez et al., 2009; Sardella and Luque, 2016). Obviously, all this indicating the necessity of the generic revision. In our opinion, forming a species complex *A. simplex sensu lato* can lead to the loss of some critical information about the individual characteristics of the biology of parasites, and opportunities to influence the changes taking place in their ethology, pathogenesis, evolution, and divergence. A solid and clear taxonomic framework is necessary for examine the basic biology of the parasites, and establish the control system in epidemiology and medicine. Thus, the scope of this study was to accurately identify species of nematodes found in minced salmon *O. nerka* using the molecular genetics methods and reconstruct phylogenetic relationships within the family Anisakidae which was originally described by Skrjabin and Karokhin (1945) and Skrjabin and Mozgovoy (1973). In the present manuscript, according to Jägerskiöld (1894) we restore the validity of the genus *Peritrachelius* (Diesing, 1851) and according to Mozgovoy (1951) upgrade subgenus *Skrjabinisakis* to genus.

## Materials and methods

### Parasite material

Minced sockeye salmon (frozen) from the Kamchatka Peninsula in the amount of 1 kg was bought at the Vladivostok’s fish market. The organoleptic properties of the product were compromised: among the muscle fibers, inclusions of intestines and membranes were identified during the visual examination of the minced fish. Using a binocular microscope (Micromed MC2 Zoom 1CR) 12 worms (third-stage larvae of nematodes) were extracted and washed in distilled water, then fixed in 70% ethanol. The size of worms ranged from 5 to 15 mm.

### PCR amplification

The total DNA was extracted using a QIAamp DNA Mini Kit (QIAGEN, Germany). The ITS1-5,8S-ITS2 region of rDNA was amplified using the classical polymerase chain reaction (PCR) method with specific primers 5′-CCGGGCAAAAGTCGTAACAA-3′ (AscITF) and 5′-ATATGCTTAAATTCAGCGGGT-3′ (R) (Naoki et al., 2010) and DreamTaq Green Master Mix (Thermo Scientific, Lithuania). Cycling conditions consist of a preliminary denaturation at 94°C for 2 min, followed by 30 cycles of denaturation at 94°C for 2 min, annealing at 49°C for 30 sec, elongation at 72°C for 1.5 min, and a final product extension at 72°C for 5 min. The amplification products were visualized on 1.5% agarose gel by ethidium bromide. Amplicons purification was conducted using exonuclease and alkaline phosphotase 1:3 (ExoSap-IT, Thermo Scientific). The PCR products were sequenced by the Sanger method using BigDye Terminator Cycle Sequencing Kit, and specific primers the same as for PCR. After the BigDye Terminator sequencing reaction products were cleaned to remove excess unlabeled dideoxynucleotides using 0.125 М EDTA, CH3COONa, and 96% C2H5OH. The nucleotide sequences were analyzed on an automatic ABI PRIZM 3130 gene analyzer (the center for collective use of FSC EATB FEB RAS). Assembly and alignment of the ITS1-5.8S-ITS rDNA sequences with similar nematode sequences from Genbank (Table 1) were performed in programs FinchTV (Geospiza Inc., Seattle, WA, USA) and MEGA7 (Kumar et al., 2016). Contiguous ITS1-5.8S-ITS2 sequences 863 bp in length were submitted to GenBank NCBI under accession numbers: MT192598, MT192599, MT250915, and MT250916. Genetic distances (p-distance; %) between sequences were calculated using the two-parameter Kimura model and the gamma distribution (0.5).

**Table 1.** Sequences of the ITS1-5.8S-ITS2 rDNA region from GenBank used in this study.

Accession number	Species	Geographical region
EU718471	*Anisakis simplex*	Morocco
JQ934875		Croatia
KJ011481		South Korea
KM273043		Denmark
KP645361		Poland
MF668920		USA
MN726484		Japan
GQ169364		Ireland
MH211473	*A. pegreffii*	China
KF032056	*A. simplex* × *A. pegreffii*	
KF032058			Turkey
KF032060		
KY524216	*A. berlandi*	Indonesia
JQ912692	*A. nascettii*	Italy
JX486104		Brazil
JN005767	*A. ziphidarum*	Portugal
EU718473		Mauritius
KF673776	*A. typica*	China
EU327689		Brazil
KX098561		USA
JQ912694	*A. brevispiculata*	Italy
EU327691	*A. physeteris*	Brazil
GU295976	*A. paggiae*	Greenland
KC970082	*Pseudoterranova cattani*	Argentina
KM273078	*P. decipiens*	Denmark
AB576757	*P. azarasi*	Japan
KM491173	*Contracaecum osculatum*	Denmark
EU678869	*C. rudolphii*	Italy
JF424598	*C. bioccai*	USA
LC422643	*Ascaris lumbricoides*	Japan

The phylogenetic relationships between nematodes were reconstructed using Neighbor-Joining (NJ) method (Kimura 2-parameter + G) in program MEGA7 (Kumar et al., 2016) and maximum likelihood (ML) method based on the Hasegawa-Kishino-Yano model (chosen using the information criteria of Bayes (BIC)) in PhyML (Guindon et al., 2010) implemented on the ATGC online bioinformatics platform. The tree with the highest log likelihood was shown. Initial trees for the heuristic search were obtained by applying the BioNJ. A Gamma shape parameter was used to model evolutionary rate differences among sites (5 categories (+G, parameter = 0.8895)). The analysis involved 34 nucleotide sequences. There were a total of 877 positions in the final dataset. *Ascaris lumbricoides* (Linnaeus, 1758) from the family Ascarididae Baird, 1853 was used as an outgroup.

## Results and discussion

Morphological analysis of the nematodes according to Mozgovoy (1953) showed that they are from the genus *Anisakis*. Molecular analysis detected that these nematodes belonged to the species *A. simplex*. All 12 sequences were identical. No fixed substitutions were found when compared nucleotide sequences of the ITS1-5.8S-ITS2 rDNA region of the studied samples with sequences of this species from different geographical localities (Table 1). Probably, the methods of nematode expansion are so effective that panmixia occurs at the whole-species level, which can be indirectly associated with anthropopression. *A. simplex* worms have the potential to affect fishing industries, which maintain food and economic stability. The detection of *A. simplex* helminths in minced fish increases the probability of infection of the human population, so there should be tight control over the distribution of such products in the food markets (Bao et al., 2017). Dead *A. simplex* larvae can save allergenic properties even after prolonged storage in frozen form (−20 ± 2°C for 11 months) (Rodríguez-Mahillo et al., 2010). WHO recognized *A. simplex* as the parasite with the largest number of known allergens 14 ‘Ani s’ proteins, demonstrated strong cross-reactivity (especially Ani s 2 (paramyosin) and Ani s 3 (tropomyosin)) to homologous proteins of other nematodes and invertebrates and resistant to pepsin (Aibinu et al., 2019; Baird et al., 2014).

**Figure 1:**
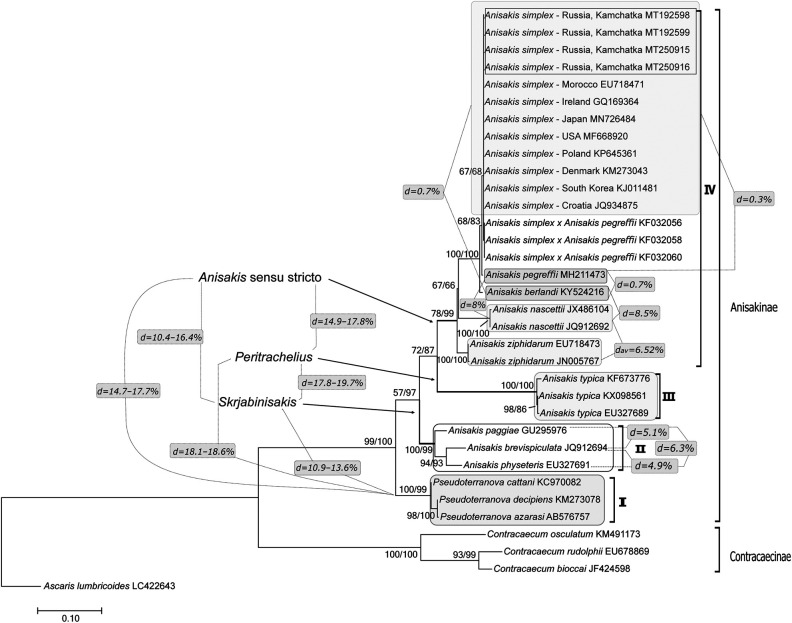
Phylogenetic relationships of family Anisakidae based on ITS1-5.8S-ITS2 rDNA sequences reconstructed by Maximum Likelihood (ML) and Neighbor-Joining (NJ) methods. Nodal support values are shown based on 1,000 bootstrap replicates (ML/NJ). The tree was drawn to scale, with branch lengths measured in the number of substitutions per site. Accession numbers are given for each species/strain at the end of each sequence. The arrows with generic names are indicating branches. Genetic distances between species and genera are indicated at the junction of the dotted lines. The scale bar represents the number of substitutions per site.

In order to establish phylogenetic relationships among anisakids, the phylogenetic tree was reconstructed based on the sequences of the ITS1-5.8S-ITS2 region with indicated ranges of genetic p-distances (Fig. 1). Two clades according to the subfamilies Contracaecinae (Anisakinae Railliet and Henry, 1912; Mozgovoy and Shakhmatova, 1971) of the family Anisakidae were distinctly distinguished on the tree. Contracaecinae clade contains three species: *Contracaecum osculatum* (Rudolphi, 1802), *C. rudolphii* (Hartwich, 1964), and *C. bioccai* (Mattiucci et al., 2008).

The Anisakinae clade branched into four distinct, reliably supported subclades. The first subclade (I) includes species of the genus *Pseudoterranova* (Mozgovoy, 1950). The species *A. paggiae* (Mattiucci, Nascetti, Dailey, Webb, Barros, Cianchi, and Bullini, 2005), *A. brevispiculata* (Dollfus, 1966), *A. physeteris* form the second subclade (II). The third subclade (III) is represented exclusively by the species *A. typica*. The species *A. simplex*, *A. pegreffii*, *A. berlandi*, *A. nascettii* (Mattiucci, Paoletti, and Webb, 2009), *A. ziphidarum* (Paggi, Nascetti, Webb, Mattiucci, Cianchi, and Bullini, 1998) make up the fourth subclade (IV). Within the IV subclade, nematodes are divided into three groups located on separate, well supported branches: (i) *A. simplex*, hybrid *A. simplex*×*A. pegreffii*, *A. pegreffii*, and *A. berlandi*; (ii) *A. nascetti* group; (iii) *A. ziphidarum* group.

When determine sibling species *A. simplex, A. pegreffii*, *A. berlandi* into a single complex *A. simplex sensu lato* Mattiucci et al. were based on analyses of data collected from allozyme loci (Mattiucci et al., 1986, 2005; Nascetti et al., 1983, 1986; Paggi et al., 1998). Probably, we should once again check the correctness of the assumption that the data on the polymorphism of enzyme loci obtained by electrophoretic methods can be considered typical for the genotype as a whole. Apparently, we are not able to estimate the overall frequency of polymorphism by simply extrapolating the data on the frequency of proteins polymorphism. To choose appropriate gene regions for resolving a particular systematic question among the organisms at a certain categorical level is still a difficult process. Such highly conserved nuclear markers as 18S and 28S rRNA genes are not able to divide the pure specimens of anisakids; moreover, there are not enough data on these markers to reconstruct the phylogenetic relationships of these worms between genera and within the family Anisakidae (Nadler et al., 2005, 2007). On the contrary, high copy and short transcribed rDNA spacers ITS1, ITS2 (Campbell et al., 1994; Hoste et al., 1995; Samson-Himmelstjerna et al., 1997) and the region spanning the ITS1, the 5.8S gene, and the ITS2 of the ribosomal DNA are suitable genetic markers for the identification of nematodes, in particular anisakid species regardless of their stage of development (D’Amelio et al., 2000; Jabbar et al., 2012, 2013). The genetic differentiation among cryptic species of the *A. simplex* complex is detectable in the ITS region of the rDNA, and this differentiation supports the validity of these species. According to our data, between the sequences of *A. simplex* and the hybrid form *A. simplex* × *A. pegreffii*, and similarly between *A. pegreffii* and *A. simplex* × *A. pegreffii*, genetic p-distances were the same – 0.1%. Genetic p-distances between the ITS1-5.8S-ITS2 regions of *A. simplex* and *A. pegreffii* were 0.3%. Genetic p-distances between *A. simplex* and *A. berlandi* (former *A. simplex* C), as well as between *A. pegreffii* and *A. berlandi* were 0.7%. Similar p-distances (0.2-0.6%) have been obtained by comparing the ITS sequences of the *A. suum* (Goeze, 1782) and *A. lumbricoides* – widely known pig parasites in China (Li et al., 2017). The researchers noted that there was no clear clustering on a phylogenetic tree reconstructed for ascarids between *A. suum* and *A. lumbricoides*. However, these worms were considered as the valid ones. The division of the *A. suum* and *A. lumbricoides* species has been a crucial factor in understanding the epidemiology of helminths and the possibility of developing methods for controlling parasitic infections for medicine and veterinary. The phylogenetic reconstruction by Tunya et al. (2020) showed a clear division of *Anisakis* spp. despite the fact that the distances between *A. pegreffii* and *A. simplex* were even less (0.1%) than those described in our study. The percentages of genetic differences vary within different worm (and other organisms’) taxa and there is no absolute ‘yardstick’ (Blasco-Costa et al., 2016).

First, we suggest that distances of 0.3 to 0.7% correspond to interspecific ranges in the structure of the family Anisakidae. Second, a powerful argument for the reconsideration of taxonomic structure of *A. simplex* species complex was phylogenetic analysis performed using the mitogenome sequences of *A. pegreffii*, *A. simplex sensu stricto*, and *A. berlandi*. Three sibling species were distinctly separated from each other and this was also strongly supported by bootstrap values (Yamada et al., 2017). Third, even gene loci coding metallopeptidase enables detection of fixed nucleotide positions (SNP) demonstration that *A. pegreffii*, *A. simplex* (s. s.), and *A. berlandi* are independent (Palomba et al., 2020). Fourth, biology of helminths cannot be the reason for determine *A. simplex* and *A. pegreffii* into one species complex. In the in vivo and in vitro studies *A. simplex sensu stricto* and *A. pegreffii* species had a differential pathogenic potential and the propensity to trigger allergic reactions (D’Amelio et al., 2020). *A. simplex sensu stricto* and *A. pegreffii* are able of hybridizing in the sympatric areas and co-infect the same fish host, but no fertile adult hybrids F1 *A. simplex* × *A. pegreffii* have been found (Aibinu et al., 2019; Mattiucci et al., 2016; Mladineo et al., 2017). Fifth, *A. simplex* and *A. pegreffii* were considered independent species by Mozgovoy (1953). So, at present, we suppose, that there is absolutely no reason to save the species complex *Anisakis simplex sensu lato*, because only the precise identification of parasites is essential for their distribution and epidemiology.

Interspecific relationships within the subclade II vary in the range of 4.9 to 6.2%, overlapping with genetic p-distances within species from the subclade IV – 0.3 to 8.0%. Pairwise comparison between species of these two subclades demonstrated a higher level of divergence more likely corresponded to the intergeneric ones – 10.4 to 16.4% (Fig. 1). This is also confirmed by the similar values of p-distances between the anisakids from IV, III, II subclades and species from the other genus *Pseudoterranova* (subclade I), which vary in the range of 10.8 to 18.6%. In addition to genetic data, the question about the morphological differences of the third-stage larvae of the species included in subclade II has already been raised, according to it, they belong to the second morphotype that distinguishes them from the species from IV and III subclades, comprising larvae of the first morphotype (Iñiguez et al., 2009). Based on these data, we suggest restoring the taxonomic status of the subgenus *Skrjabinisakis* (Mozgovoy, 1953) upgrading it to the genus level. Thus, this subgenus was first described in the genus *Anisakis* for species *A. physeteris*, *A. skrjabini*, and *A. schupakovi* (Mozgovoy, 1953). Then after Mozgovoy subgenus *Skrjabinisakis* supported by Japanese scientists based on the morphology of adults and larva (Oishi et al., 1970). Based on larval morphology and genetic data for *A. physeteris* and later for *A. brevispiculata* published by Mattiucci et al. (1986, 2001), respectively, the validity of the subgenus *Skrjabinisakis* was justified as well. Of these worms species status was confirmed using molecular genetics data only for *A. physeteris* (Iñiguez et al., 2009).

The species *A. typica* (subclade III) does not take an unambiguous position on phylogenetic trees reconstructed using various methods with respect to the other *Anisakis* spp. (Iñiguez et al., 2009; Sardella and Luque, 2016), which may suggest paraphyletic relationships among representatives of the genus *Anisakis*. The genetic distances for *A. typica* indicate its remarkable divergence from the other species of the subfamily Anisakinae. Thus, *A. typica* is very distant from: species of subclade II (17.8-19.7%); species of subclade IV (14.9-17.8%); species of genus *Pseudoterranova* (18.1-18.6%) (Fig. 1). Based on the intergeneric distances of *Pseudoterranova* species and *A. typica*, the latter should be considered in the distinct genus. It is known from the literature that this species was previously put in the genus *Peritrachelius* (Diesing, 1851; Jägerskiöld, 1894). Until 1882, this genus has been considered separate and included the species *P. insignis* (synonym for *Anisakis insignis*) (Dräsche, 1882). *Peritrachelius* was a subgenus of *Ascaris* with species *Anisakis typica* and *Anisakis insignis* (Jägerskiöld, 1893). Later Mozgovoy (1953) synonymized *Peritrachelius* with the genus *Anisakis* (Mozgovoy, 1953). Based on the genetic data analysis, we suggest restoring the genus *Peritrachelius* for *Anisakis typica*.

To sum up, the true genus *Anisakis sensu stricto* probably includes independent species: *A. simplex sensu stricto*, *A. pegreffii*, *A. berlandi*, *A. ziphidarum*, and *A. nascettii*. In the structure of the subfamily Anisakinae, for the first time, it was proposed to restore two genera: *Skrjabinisakis* including species *A. paggiae*, *A. brevispiculata*, and *A. physeteris*, and *Peritrachelius* – for *A. typica*.

Short annotated list of some genera of the family Anisakidae (Skrjabin and Karokhin, 1945)

The genus *Anisakis* (Dujardin, 1845)

Diagnosis: Ventriculus long, S-shaped or straight (juveniles), length is larger four or more times than width. Vulva located in the midbody or anteriorly or posteriorly to it. Spicules long, its length exceeds 1.5 mm.

Type species: *Anisakis dussumierii* (Beneden, 1870) Baylis, 1920 (sensu Mozgovoy, 1953).

*Anisakis simplex* (Rudolphi, 1809) Baylis, 1920

Synonyms: *Ascaris simplex* (Rudolphi, 1809), nec. *A. simplex* (Dujardin, 1845); *Ascaris angulivalvus* (Creplin, 1851); *Anisaks salaris* (Gmelin, 1790) Yamaguti, 1935.

Hosts: *Balaenoptera acutirostrata* (Lacepede, 1804), *B. borealis* (Lesson, 1828), *B. musculus* (Linnaeus, 1758), *B. species*; *Delphinapterus leucas* (Pallas, 1776); *Delphinus delphis* (Linnaeus, 1758), *D. species*; *Eumetopias jubatus* (Schreber, 1776); *Hyperoodon rostratus* (Van Beneden and Gervais, 1880); *Lagenorhynchus albirostris* (Gray, 1846), *L. obscurus* (Gray, 1828); *Mesoplodon bidens* (Sowerby, 1804); *Monodon monoceros* (Linnaeus, 1758); *Phocaena phocaena* (Linnaeus, 1758); *Pseudoreca crassidens* (Owen, 1846); *Platanista gangetica* (Lebeck, 1801); *Globicephala melaena* (Traill, 1809); *Orcinus orca* (Linnaeus, 1758); *Stenella coeruleoalba* (Meyen, 1833); *Halichoerus grypus* (Fabricius, 1791); *Phoca vitulina* (Linnaeus, 1758).

Localization: stomach, intestine, esophagus.

Distribution: Northern waters of the Atlantic Ocean and the Pacific Ocean; Baltic Sea.

*Anisakis pegreffii* (Campana-Rouget and Biocca, 1955)

Synonym: *Anisakis simplex* A of Nascetti, Paggi, Orecchia, Smith, Matticucci, and Bullini (1986).

Hosts: *Monachus monachus* (Hermann, 1779); *Delphinus delphis* (Linnaeus, 1758); *Ziphius cavirostris* (Cuvier, 1823); *Tursiops truncates* (Montagu, 1821).

Localization: digestive tract (first part of the intestine).

Distribution: the Mediterranean Sea (type locality – East coast of Sardinia, near Dorgali) and the waters of the Southern Atlantic Ocean.

*Anisakis berlandi* (Mattiucci, Cipriani, Webb, Paoletti, Marcer, Bellisario, Gibson and Nascetti, 2014)

Synonym: *Anisakis simplex* sp. C of Mattiucci et al. (1997).

Hosts: *Pseudoreca crassidens* (Owen, 1846); *Ziphius cavirostris* (Cuvier, 1823).

Localization: stomach.

Distribution: Southern waters of the Atlantic Ocean and Northern Pacific Ocean.

*Anisakis nascettii* (Mattiucci, Paoletti and Webb, 2009)

Synonyms: *Anisakis* sp. A of Pontes et al. (2005) and Iglesias et al. (2008); *Anisakis* sp. of Valentini et al. (2006).

Host: *Mesoplodon grayi* (Von Haast, 1876).

Localization: stomach.

Type locality: New Zealand coast, South Pacific.

*Anisakis ziphidarum* (Paggi, Nascetti, Webb, Mattiucci, Cianchi and Bullini, 1998)

Hosts: *Mesoplodon mirus* (True, 1913), *M. layardii* (Gray, 1865); *Ziphius cavirostris* (Cuvier, 1823).

Localization: stomach.

Distribution: the Mediterranean Sea and the Southern waters of the Atlantic Ocean.

The genus *Skrjabinisakis* (Mozgovoy, 1951) stat. n.

Diagnosis: Ventriculus short, straight, length is equal or nearly equal to width. Vulva located in the anterior fourth or third of the body. Spicules short, its length not exceeding 0.67 mm.

Type species: *Skrjabinisakis physeteris* (Baylis, 1923) comb. n.

*Skrjabinisakis physeteris* (Baylis, 1923) comb. n.

Synonym: *Anisakis physeteris* (Baylis, 1923); *Anisakis skrjabini* (Mozgovoy, 1949).

Hosts: *Physeter catodon* (Linnaeus, 1758), *P. microcephalus* (Linnaeus, 1758); *Kogia breviceps* (Blainville, 1838); *K. sima* (Owen, 1866); *Ziphius cavirostris* (Cuvier, 1823).

Localization: stomach.

Distribution: South-Eastern Africa; the Mediterranean Sea and the Atlantic Ocean.

*Skrjabinisakis schupakovi* (Mozgovoy, 1951)

Synonym: *Anisakis* species (Schupakov, 1936).

Hosts: *Phoca caspica* (Gmelin, 1788).

Localization: stomach.

Distribution: Caspian Sea (Chechen Island).

*Skrjabinisakis paggiae* (Mattiucci, Nascetti, Dailey, Webb, Barros, Cianchi and Bullini, 2005) comb. n.

Synonyms: *Anisakis paggiae* (Mattiucci, Nascetti, Dailey, Webb, Barros, Cianchi and Bullini, 2005).

Hosts: *Kogia breviceps* (Blainville, 1838); *K. sima* (Owen, 1866).

Localization: stomach.

Distribution: the Atlantic Ocean, particularly in its central part.

*Skrjabinisakis brevispiculata* (Dollfus, 1966) comb. n.

Synonyms: *Anisakis brevispiculata* (Dollfus, 1966).

Hosts: *Kogia breviceps* (Blainville, 1838); *K. sima* (Owen, 1866); *Physeter microcephalus* (Linnaeus, 1758).

Distribution: the Mediterranean Sea and the waters of the Central Atlantic Ocean.

Localization: stomach.

The genus *Peritrachelius* (Diesing, 1851)

Diagnosis (sensu Stiles and Hassall, 1899): Ventriculus medium-sized, S-shaped, length is larger nearly five times than width. Vulva located near the midbody. Spicules unequal, long: left – 3.0 mm, right – 0.9 mm length.

Type species: *Peritrachelius typicus* (Diesing, 1860; Jägerskiöld, 1894).

*Peritrachelius typicus* (Diesing, 1860; Jägerskiöld, 1894)

Synonyms: *Anisakis alexandri* (Hsü and Hoeppli, 1933); *Anisakis tursiopis* (Crusz, 1946); *Conocephalus typicus* (Diesing, 1860); *Anisakis typica* (Baylis, 1920; Diesing, 1860).

Hosts: *Delphinus delphis* (Linnaeus, 1758); *Globicephalus melas* (Flower, 1885); *Phocaena phocaena* (Linnaeus, 1758); *Phoca* sp.; *Lagenorhynchus obscurus* (Gray, 1828); *Sotalia fluviatiles* (Gervais and Deville, 1853); *Stenella coeruleoalba* (Meyen, 1833), *S. attenuate* (Gray, 1846), *S. longirostris* (Gray, 1828); *Steno bredanensis* G. Cuvier in Lesson, 1828; *Lagenodelphis hosei* (Fraser, 1956).

Distribution: Germany, South-Eastern Africa; warm waters of the Atlantic Ocean.

Localization: stomach.
